# A fatal fecaloma

**DOI:** 10.1186/s12245-020-00305-w

**Published:** 2020-08-20

**Authors:** Elsa Logre, Lauriane Degravi, Gaëtan Plantefève, Damien Contou

**Affiliations:** Service de Réanimation Polyvalente, Centre Hospitalier Victor Dupouy, 69 Rue du Lieutenant-Colonel Prudhon, 95100 Argenteuil, France

## Abstract

**Background:**

Fecal impaction may complicate chronic constipation. We report a fatal case of fecal impaction in a patient treated with long-term neuroleptic treatment.

**Case presentation:**

A 70-year-old man with a history of severe chronic psychosis treated with olanzapine was admitted to the emergency department for acute abdominal pain and increased abdominal perimeter. Abdominal computed tomography revealed a severe fecal impaction with no sign of peritonitis or acute mesenteric ischemia. The patient eventually died from multi-organ failure 2 days after his admission to the intensive care unit.

**Conclusions:**

Chronic constipation with fecal impaction is a well-known complication of long-term neuroleptic treatment. Severe forms may be life-threatening. Prevention with systematic administration of laxatives appears of paramount importance.

## Case synopsis

**Fig. 1:**
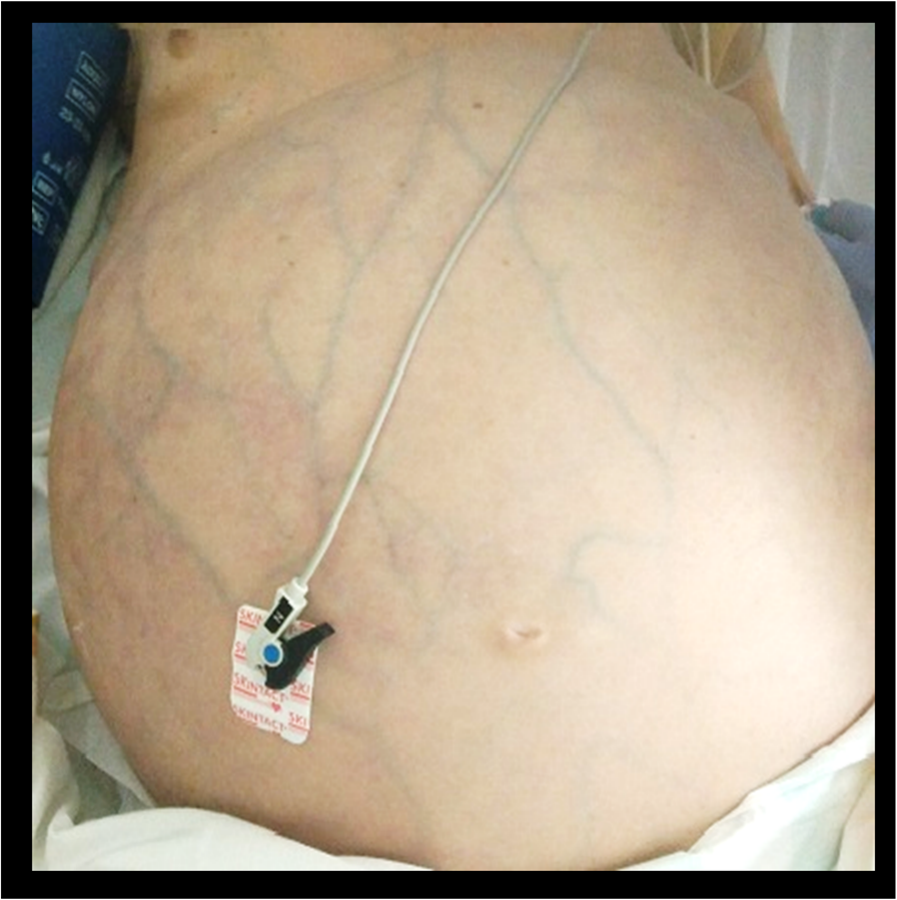
Abdominal distension and diffuse collateral venous circulation

A 70-year-old man with a history of severe chronic psychosis treated with olanzapine was admitted to the emergency department for acute abdominal pain. He reported the absence of stools for 3 weeks together with an increasing abdominal perimeter. The patient developed severe arterial hypotension requiring ICU admission for vasopressor support. Upon ICU admission, the patient had a severely distended abdomen with a diffuse collateral venous circulation (Fig. 1). There was no clinical sign of ascites nor spider angioma. Laboratory tests revealed hyperlactatemia (6.7 mmol/L, *N* < 1.6 mmol/L) and acute kidney failure (creatinine 185 μmol/L, *N* < 110 μmol/L) with anuria.

## Diagnosis

**Fig. 2:**
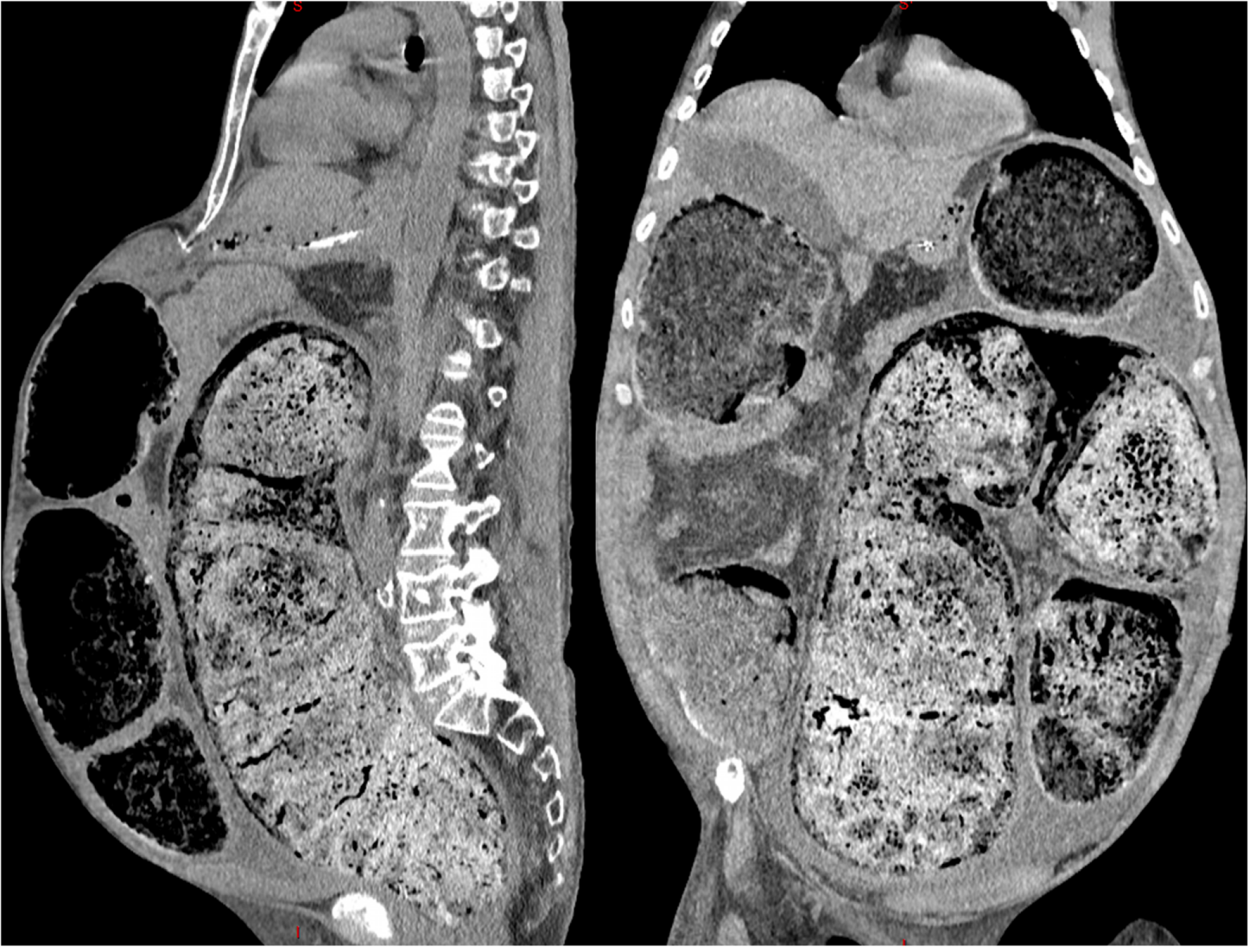
Saggital (left) and coronal (right) views of the abdominal computed tomography revealing a severe fecal impaction

**Fig. 3:**
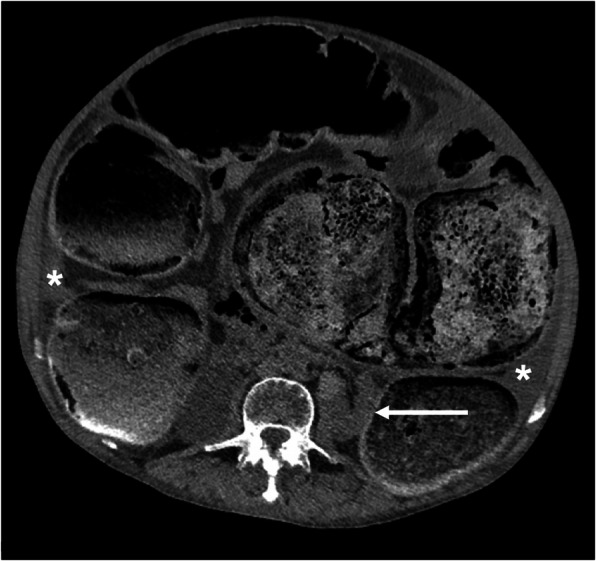
Axial view of the abdominal computed tomography revealing a marked backward compression of the kidneys (white arrow) together with aperitoneal effusion (***)

Abdominal computed tomography depicted a severe fecal impaction (Fig. 2) with a marked backward compression of the kidneys (Fig. 3, white arrow) together with a peritoneal effusion (Fig. 3, ***). There were no signs of pneumoperitoneum, bowel pneumatosis, or parietal thickening. Because of the previous limited autonomy of the patient with cognitive decline, a conservative strategy without surgical extraction of the fecaloma was decided. The patient eventually died on day 2.

Chronic constipation with fecal impaction is a well-known complication of long-term neuroleptic treatment [1, 2]. Severe forms may be life-threatening [3, 4] and may require an emergency laparotomy [5] before the onset of intraabdominal complications. In this context, prevention with systematic administration of laxatives appears of paramount importance.

## Data Availability

Relevant data and supporting material are available on request.
